# The role of community assets in tackling UK health inequalities through community engagement and partnerships in social prescribing

**DOI:** 10.3389/fpubh.2026.1761446

**Published:** 2026-03-11

**Authors:** Linda J. M. Thomson, Helen J. Chatterjee

**Affiliations:** Culture, Nature, and Health Research Group, UCL Arts and Sciences/UCL Biosciences, University College London, London, United Kingdom

**Keywords:** community assets, health inequalities, link worker, mental health, partnerships, peer support, self-determination theory, social determinants of health

## Abstract

**Introduction:**

With policy focused on prevention and re-location of care into communities to reduce the burden on acute services, the need for robust evidence from social prescribing that integrates community assets into health and care systems is becoming increasingly vital.

**Methods:**

As part of a national multi-centered program of research, ‘Mobilizing Community Assets to Tackle Health Inequalities’, seven projects from across the UK participated in an online survey asking how their work involved social prescribing, successes and challenges, and tackling health inequalities. Survey data was supplemented with information from quarterly spreadsheets where projects recorded academic and creative outputs, events, partnerships, posts, ways of working, and audiences or communities.

**Results:**

Six themes: activities/interventions; audiences/communities; funding; methods; partners/stakeholders; and research aims/focus, with 62 subthemes were derived from the survey data. Responses showed that projects worked in the UK’s most deprived areas delivering social prescribing focusing on the social determinants of health, and mapping community assets and pathways for underserved groups. Successes were attributed to link workers as knowledge brokers, and partnerships with primary and secondary care, and arts and community organizations in co-producing local interventions. Challenges such as variation in the flexibility of creative approaches and lack of knowledge about community assets were also attributed to link workers. Other challenges included accessibility, participant non-attendance, and funding issues.

**Discussion:**

To tackle health inequalities, projects used hyper-local, place-based approaches and co-design of interventions promoting cultural and green community assets, with link workers, people with lived experience, peer support workers and volunteers. The study proposes an approach to social prescribing that combines models of recovery and peer support underpinned by self-determination theory to improve social inclusion and quality of life. Recommendations include a consortium-based approach to person-centered care working closely with local populations and public health, where provision is co-located and co-delivered in conjunction with relevant data concerning health conditions and the wider social determinants to address the root causes of health inequalities.

## 1 Introduction

“Mobilizing Community Assets to Tackle Health Inequalities” (Mobilizing Community Assets) is a three-phase UK-wide interdisciplinary and multi-centered program of research funded by UKRI-AHRC and coordinated by a national, university-based team. In phase one 12 projects were funded for 12 months (January–December 2022) to investigate how community partnerships and community, cultural, and natural assets could be used to improve mental and physical health outcomes, in phase two 16 projects were funded for 9 months (November 2023–July 2024) to establish cross-sector partnerships and consortia using collaborative approaches, and in phase three, 12 projects were funded for 3 years (March 2024–March 2027) to scale up place-based community consortia building projects. Several of the projects offered social prescribing to people from diverse backgrounds including adults and young people with mental illness; people in recovery and/or experiencing homelessness and social isolation; ethnic, refugee and migrant communities; and people with dementia. Projects were located in urban, rural and remote locations and in specific regions. The study examined how projects used university, community and healthcare partnerships to tackle health inequalities through social prescribing approaches using cultural and green community assets to deliver arts and heritage activities. The Mobilizing Community Assets program was specifically set up to find effective ways to tackle health inequalities across the UK through collaboration and consortia building projects. Asset-based working is increasingly recognized as a valuable approach for tackling health inequalities (1–3).

An expert opinion consultancy process, comprising an anonymous online survey and a consultation workshop, commissioned to identify future research priorities to address UK societal and structural health inequalities (1), demonstrated a clear need to assess the impact of cultural and natural assets on reducing inequality. Findings demonstrated that communities could be improved through a sense of place, urban regeneration and tackling issues such as homelessness and substance abuse but that the research base was patchy and longitudinal studies were needed (1). Asset-based initiatives in Valencia, Spain and Sheffield, UK to train local volunteers to become health promoters in less-advantaged neighborhoods identified three main processes associated with change: “enabling asset-based thinking,” “developing asset-based capacities,” and “changing decision-making and wider health determinants through asset-based approaches” [(2), p. 4]. Asset-based capacities involved developing relationships where people were valued for their skills and expertise rather than looking for deficits but a lack of dedicated time and funding for partnership work countered the potential impact of community assets (2). Other researchers also advocated asset-based, salutogenic approaches to promote self-esteem and the coping abilities of individuals and communities, eventually resulting in less dependency on professional services (3). The authors proposed that redressing the balance between asset-based and deficit-based approaches in public health could remove barriers to addressing health inequalities (3). Health inequalities are defined as “systematic, unfair and avoidable differences in health across the population, and between different groups within society” which affect mental and physical health and wellbeing [(4), p. 2]. The term denotes “differences in health across subgroups of the population according to social class, sex, education, income or wealth, race or ethnicity, or geography” [(5), p. i501] and refers to the quality of built and natural environments in which people live and work (6). Furthermore, different groups have varying experiences of conditions, social and psychological support, and behavioral options rendering them “more or less vulnerable to poor health” [(6), p. 3]. Tackling health inequalities, therefore, implicates the social determinants of health; the conditions in which “people are born, grow, live, work, and age” [(7), p. 40]. In the UK there is a widening gap in life expectancy where England remains healthier than the devolved nations of Wales, Scotland and Northern Ireland (5). In England, however, there are sizeable disparities between regions in the North with worse health and a lower life expectancy of around 2 years than in the South (5). For instance, analysis of publicly available data over time found that people in the North East are likely to have a shorter life and spend a larger proportion of their lives in poor health, as well as being more likely to “die prematurely from preventable diseases” [(8), p. 63].

The COVID-19 pandemic occurred against the context of prevailing health inequalities resulting in an elevated risk of mortality in areas of higher deprivation, partly because of the greater incidence of people with pre-existing health conditions (5). Additional and cumulative risk factors were found among those living in deprived areas, in specific geographic regions, experiencing overcrowding or working in roles with proximity to others (9). Effects of the pandemic continue to impact population health and health inequalities in the UK. The British Medical Association observed that “millions have seen their quality of life affected by long COVID” and many people have reported poorer mental and physical health because of the pandemic. However, “none of this has been felt equally, with ethnicity, age, disability status, and other factors meaning some social groups have been more affected than others” [(10), p. 1]. Previous research highlighted the significant role that community assets played in supporting communities during the COVID-19 pandemic (11), and the impact of culture-, health-, and nature-based engagement on mitigating the adverse effects of public health restrictions on wellbeing, social connectedness and loneliness (12). A review of over 200 sources, including peer-reviewed and grey literature, identified that community initiatives sought to mitigate social, physical and psychological vulnerabilities during the pandemic with resources aimed at wellbeing and positive mental health, or social isolation and loneliness (11). As few of the studies employed validated measures with most reliant on anecdotal evidence, the impact of the initiatives was difficult to verify (11). Evidence from two surveys with almost 4,000 respondents found that frequency of engagement in culture-, health- and nature-based activities was a significant predictor of wellbeing, but type of activity was not significant (12). Furthermore, respondents who felt more connected to others, either online or in-person, reported better psychological wellbeing than those who felt less connected (12).

The National Health Service (NHS) long term plan (13) was set up with the intention of training a thousand social prescribing link workers in primary care networks to make referrals to non-medical sources of care. This plan was impacted by COVID-19 after 2 years though by this time 85 per cent of link workers had been employed. The plan was replaced by “Fit for the future: the 10 year health plan for England” (14) that proposed three shifts in the way that care was administered: from hospital to community, analogue to digital, and sickness to prevention. These changes entailed a move from hospital-centric care, seen as siloed and detached from communities, to care in a patient’s home or Neighborhood Health Centre, and in hospital only if necessary (14). Aims of the plan pertinent to the current research included tackling health inequalities, moving care into the community, and partnership and collaboration. With respect to tackling health inequalities, the 10 year health plan stated that social determinants explain the country’s wide and widening health inequalities (9) and recognized the injustice that social determinants of ill health cluster in deprived areas.

The current study considered an approach to social prescribing related to the social determinants of health (15) that combines existing models of recovery and support underpinned by self-determination theory to improve social inclusion and quality of life. Models of recovery include peer support groups, defined as “non-clinical assistance from individuals with similar conditions or circumstances to achieve long-term recovery from psychiatric, alcohol, and/or other drug-related problems” [(16), p. 143]. Allied to peer support groups, other authors stressed the importance of lived experience in models of care to aid engagement and motivation and provide monitoring (17). Self-determination theory is pertinent for the current study as it is a general theory of human motivation and personality (18) that focuses on the extent to which behavior is self-determined. The theory explicitly identifies that when people are supported, more autonomous forms of behavioral regulation occur (18). Three needs are central to the model: autonomy (feeling of being the origin of one’s own behaviors), competence (feeling effective), and relatedness (feeling understood and cared for by others) (18). A meta-analysis of self-determination theory in healthcare contexts suggested that self-determination theory is a viable conceptual framework to study outcomes of motivation for health-related behaviors, and interventions for improved health care and mental and physical health (19).

## 2 Methods

### 2.1 Design

The study conducted an online survey and extracted data from quarterly spreadsheets.

### 2.2 Participants

Principal investigators (PIs) and co-investigators (*n* = 8) from Mobilizing Community Assets projects (*n* = 7) and their project partners and stakeholders (*n* = 21) including community researchers, local council staff, link workers, peer support workers, and people with lived experiences consulted by the PIs. As 10 of the 40 Mobilizing Community Assets projects included social prescribing in their remit, the sample of seven projects (70%) that responded to the survey was reasonably representative of the program, particularly as projects were from five UK regions (Greater London; North West; Scotland; West Midlands; and Yorkshire and the Humber) and covered all three phases of the program over 4 years, with four projects participating in two phases and three projects participating in one phase (Table 1).

**Table 1:** Mobilizing community assets projects involving social prescribing.

Project	Phase(s)	UK region	Activity/intervention	Partners and stakeholders	Research aims/focus
1	Phase 1	West Midlands	Place-based green social prescribing service for people with dementia	Health professionals; people with lived experience and the voluntary sector	To establish green social prescribing through collaboration with partners, and address cross-cultural adaptations of social prescribing models
2	Phase 1	Yorkshire and the Humber	Embedding arts and culture in health systems for people living with dementia	Anchor, arts and cultural organizations; GPs; and peer support groups	To use community-based participatory research to understand the potential for expanding creative activities and access to the environment
3	Phases 1 and 3	Scottish Highlands	Arts / culture / heritage based hands-on activities for mental health referrals and social isolation	Health professionals; third sector support organizations and voluntary sector	To deliver non-pharmaceutical interventions at scale in remote and rural contexts to improve referral pathways, and reduce place-based health inequalities
4	Phases 1 and 3	North West	Matching referred young people with creative health practitioners to support mental health	Artists; therapists; health and social care professionals; mental health organizations and people with lived experience	To integrate arts therapy into NHS talking therapies to support mental health in young people and develop digital innovations to facilitate social prescribing pathways
5	Phases 2 and 3	North West	Integrating story telling into preventative health measures such as cancer screening	Community researchers; peer support groups and link workers	To encourage community engagement and partnerships in social prescribing, and tackle health inequalities in a hyper-localized way
6	Phases 2 and 3	Scottish Lowlands	Mapping social prescribing pathways for people in recovery and/or homeless	Health and social care professionals; peer support groups; and third and voluntary sectors	To explore links between creativity, relationships, nature and health, and to develop systems that promote equitable health and wellbeing
7	Phase 3	Greater London	Interventions to promote inclusion of cultural and green community assets in social prescribing	Community organizations; health professionals; link workers; people with lived experience and voluntary sector	To enable access to green spaces for Black, minority ethnic and refugee communities to improve mental health and wellbeing and address social justice

### 2.3 Materials

Materials consisted of the participant information and consent form, a Microsoft Forms online survey with four main questions (see section 3.2) and five quarterly spreadsheets where projects recorded their academic and creative outputs, events, partners, and the involvement of communities and people with lived experience.

### 2.4 Procedure

The survey was conducted (July–October 2025) using purposive sampling and self-selection with PIs of projects involving social prescribing from three phases of the program. A link to the survey was placed in the chat of online meetings and emailed to PIs who were advised to consult partners and other stakeholders to complete the questions and write as fully as possible; responses were summarized (Table 2). Respondents submitted 4,500 words and took on average 54 min to complete the survey. The survey was supplemented with data from quarterly spreadsheets (May 2024–August 2025) completed by PIs on a mandatory basis contingent on acceptance of their awards.

**Table 2:** Mobilizing community assets project summaries of responses to survey.

Project	How the project involves social prescribing	Social prescribing successes and how these came about	Social prescribing challenges and how these were addressed	Extent to which social prescribing is tackling health inequalities
1	Delivering green social prescribing over several years focusing on the wider social determinants of health in one of the UK’s most deprived wards as a direct response to community needs.	Using green social prescribing to engage and build trust as well as self-management even alongside clinical treatment has made a positive difference to local communities.	Local spaces and allotments do not feel accessible for people who are newly settled or do not speak English as a first language. These issues are being addressed through partnership working.	Organizational commitment and leadership are centered around tackling health inequalities at the root, work which is underpinned by Marmot principles.
2	Referrals from GPs, memory support workers and social prescribers are received by the adult social care service that supports people with dementia to engage in creative and cultural activities.	Engaging people with dementia in co-creating a theater festival and forming a new choir through partnering with other arts organizations to create more accessible routes.	Being a high-profile arts organization attracts people from a broader geographical area, but as referrals are only possible within a localized area due to limited funding, which is always an issue.	Referrers open up a path to a free creative opportunity for people from marginalized or lower socio-economic backgrounds as financial barriers should not arise when people are feeling most vulnerable.
3	Scaling up a social prescribing intervention used with students and delivering it in rural communities to adults referred by healthcare professionals or third sector support organizations.	There was a great deal of interest in and support for the intervention that addressed geographic and logistical challenges such as accessibility and lack of public transport.	Support for the intervention was not translated into widespread referrals and take-up of places due to variable referral pathways, volunteer recruitment and perception of the heritage sector.	Exploring how rural health inequalities are measured and how participation in heritage and engagement with local assets can be widened amongst more disadvantaged groups in the community.
4	Co-investigators are involved in social prescribing through direct delivery and system level development aimed at improving access to meaningful creative health opportunities for young people.	Uncovering direct stories of impact from across the region, embedding creative health offers within place-based organizations, and different routes and referral pathways for young people.	Many young people face significant personal, emotional, and structural barriers limiting participation; non-attendance is often linked to anxiety, depression, or low motivation.	To integrate arts therapy into NHS talking therapies to support mental health in young people and develop digital innovations to facilitate social prescribing pathways.
5	Social prescribers sit on our hyper-localized teams, and we work closely with link workers on a regular basis though their ability to engage depends on their workload.	Engaging with link workers and building trust as they can often bridge the gap to translate a health message into an accessible language to a community.	Case load and level of freedom of link workers to engage in creative projects can vary depending on the nature of the organization through which they are line managed.	An essential part of tackling health inequalities is helping to build communities to convene and support themselves from within.
6	Collecting stories of people navigating health systems and identifying gaps needed for effective social prescribing to gain a better understanding of how to connect individuals to community-based opportunities for health and wellbeing.	Forming ties with community members, developing trusting relationships with organizations working with people in recovery and/or experiencing homelessness, and getting information out to people who will really benefit.	Knowledge gap about what is available in terms of support from community assets combined with unknown pathways to make referrals is a factor that could restrict access to services for people experiencing diverse vulnerabilities.	Using a place-based approach to understand how policy makers, researchers, people with lived experience, health and third sectors can play a role in integrating community assets and social prescription.
7	Interviews conducted with social prescribers and link workers found that engagement with community assets is lower among ethnic minority and refugee communities yet can offer both prevention and management of symptoms of mild to moderate mental illness.	Benefits of locating social prescribing in community centers rather than in GP surgeries as many clients, especially young people, struggle to engage with social prescribing based in surgeries, associating the spaces with clinical problems.	Lack of integration of the local asset ecosystem into social prescribing, stigma associated with mental health among some ethnic minority and refugee communities, and lack of understanding of the benefits of engaging with community assets.	Knowledge generated through workshops, focus groups, community walks and interviews is being used to co-design interventions to promote inclusion of community assets in social prescribing to address ethnic inequalities in mental health.

### 2.5 Analysis

Survey data were analyzed in Lumivero NVivo 14 (20) using thematic analysis (21). Inductive thematic analysis was used to determine themes within the context of social prescribing and deductive thematic analysis was used to examine successes and challenges to which respondents referred. The derivation of themes and subthemes was conducted by one researcher and checked by another with any queries resolved through further discussion. Data analysis was informed by an interpretivist epistemological perspective employing a constructivist methodology to understand respondents’ responses to the survey questions. Descriptive statistics were used to interpret information from the quarterly spreadsheets.

## 3 Results

Six themes: activities/interventions; audiences/communities; funding; methods; partners/stakeholders, and research aims/focus, with 62 subthemes and typical quotations were derived from the survey data (Table 3). Quarterly spreadsheets showed that each project on average produced two academic and four creative outputs; held two events each attended by between 200 and 250 delegates, had 30–40 partners, created six new posts (4 full time equivalent), and involved two target communities and up to 100 people with lived experience.

**Table 3:** Themes, subthemes (highest to lowest number) and examples of typical quotations.

Themes (no. of subthemes)	Subthemes	Examples of quotations
Methods (15)	Case studies	“Case studies of successful interventions involving social prescribing will be developed”
	Collecting stories of people navigating local health systems	“Stories of people navigating health systems and the identification of gaps”
	Co-creation, co-design, co-production, and co-participation	“We are taking on board these challenges in the co-design of interventions”
	Ethnographic observation and video reflective ethnography	“We are using ethnography observation, video reflective ethnography…”
	Focus groups	“Knowledge generated from our workshops, focus groups, community walks and interviews”
	Knowledge exchange events	“We are using… knowledge exchange events designed/conducted with social care practitioners”
	Lived experience workshops	“To date, we have undertaken a cycle of 12 collaborative lived experience workshops…”
	Mapping community assets	“Working with stakeholders to map community assets as communities see them”
	Mapping social prescribing pathways	“A new map on social prescribing pathways has been developed”
	Marmot principles	“Our work is underpinned by the Marmot principles”
	Measuring wellbeing	“Over 500 positive outcomes using the Warwick and Edinburgh wellbeing scale”
	Mixed methods	“Our mixed-methods place-based project is being undertaken with five ethnic minority and refugee community organizations”
	Place-based approaches	“Frameworks that help young people navigate and access place-based creative opportunities”
	Questionnaires	“Questionnaires designed and conducted with … people in recovery and with lived experience of homelessness”
	Semi-structured interviews	“We are using… focus groups designed/conducted with… people in recovery”
Partners/stakeholders (15)	Community embedded researcher	As a community embedded researcher… I’ve been invited to join the social prescribing network”
	Community psychiatric (CPNSs) and drug and alcohol nurses	“We work with … CPNSs and drug and alcohol nurses, who are all active in referring people”
	Community voice champions	“Participants include volunteer community voice champions recruited by community organizations”
	General practitioners (GPs)	“Referrals from GPs… are received initially by the social care service who in turn refer…”
	Health and social care practitioners	“Questionnaires designed and conducted with health and social care practitioners”
	Link workers	“Frontline link workers further reduce inequality by offering early, relational support to young people”
	Local authorities	“The next phase of our work involves semi-structured interviews with social prescriber decision-makers working for local authorities”
	Memory support workers	“Referrals from… memory support workers… are received initially by the social care service”
	Mental health practitioners	“Mental health practitioners can refer young people directly to the… team, who then match them with a creative partner”
	National Health Service	“High-quality creative opportunities become more visible, discoverable, and consistently connected to referral routes through the NHS…”
	Peer support service	“We have worked in partnership with the peer support service - an adult social care service for people living with dementia for the past 15 years”
	People with lived experience	“To date, we have undertaken a cycle of 12 collaborative lived experience workshops… with 27 community voice champions”
	Policy makers	“Oral health improvement program for people experiencing homelessness… collaboration between university, NHS, policy makers…”
	Third sector organizations	“Third sectors can play a role in the strengthening and integration of community assets
	Voluntary sector organizations	“Our work involves semi-structured interviews with… the charity and voluntary sector”
Activities/interventions (11)	Arts activities	“Aim was to deliver arts based hands-on activities for adults experiencing mild mental health conditions and social isolation”
	Cancer screening	“Tackling health inequity in a hyper- localized way on topics such as… Cancer screening…”
	Civic engagement	“Integration of community assets, social prescription and civic engagement for people who are often excluded from the system”
	Community walks	“Knowledge generated from our… community walks and interviews are being used to co-design… interventions”
	Cultural activities	“To enhance cultural and green inclusion in social prescribing… to address ethnic inequalities in mental health”
	Engagement with local assets	“Exploring how… engagement with local assets can be widened amongst more disadvantaged groups in the community”
	Green activities in natural environments	“Nature and arts play a role in the improvement of general health through social prescription”
	Heritage activities	“Exploring how participation in heritage… can be widened amongst more disadvantaged groups in the community”
	Lived experience workshops	“We have undertaken a cycle of 12 collaborative lived experience workshops…”
	Theater activities	“To create more accessible routes for people with dementia to engage with cultural offer of the city in the theater, museums, galleries and libraries”
	Workshops	“Partners getting their information out to people who they think would really benefit from our work and having them come along to workshops”
Research aims/focus (8)	Developing trusted relationships with organizations	“The first stage of our work was to develop trust relationships with organizations in Scotland working with people in recovery”
	Identifying gaps in social prescribing provision	“The identification of gaps needed for effective social prescribing has been collected”
	Mapping social prescribing pathways	“A new map on social prescribing pathways has been developed”
	Measuring health inequalities	“This phase is looking at how rural health inequalities are measured”
	Tackling health inequalities in a hyper-localized way	“Social prescribers sit on our hyper-localized teams, and we work closely with link workers on a regular basis”
	Widening participation in heritage	“To explore how participation in heritage can be widened amongst more disadvantaged groups”
	Widening engagement with local assets	“Engagement with local assets can be widened amongst more disadvantaged groups in the community”
	Wider social determinants of health	“We have been delivering on social prescribing for a number of years focusing on the wider social determinants of health”
Audiences/communities (10)	Ethnic minority groups	“Interventions that promote the inclusion of community assets in social prescribing to address ethnic inequalities in mental health”
	Migrant and refugee communities	“Refugee communities in England tend to live in unhealthy urban environments and are at greatest risk of poor mental health and wellbeing”
	People excluded from the system	“Integration of community assets, social prescription and civic engagement for people who are often excluded from the system”
	People experiencing homelessness	“A six-week program for people in recovery and/or experiencing homelessness was co-designed with these key stakeholders”
	People experiencing social isolation	“Many participants face overlapping disadvantages, including… social isolation and long waits for formal services”
	People in recovery	“Research to map social prescribing pathways for those people in recovery”
	People living in the most deprived wards	“The wellbeing center sits in one of the most deprived wards in the UK”
	People living in rural and remote areas	“Focused on the challenges of scaling up and delivering social prescribing in rural areas”
	People living with dementia	“Referrals … are received initially by the social care service who in turn refer and support people diagnosed with dementia”
	People with mental health conditions	“Adults experiencing mild mental health conditions who had been referred by healthcare professionals”
Funding (3)	Obtaining funding	“Funding, of course, if always an issue for arts organizations anyway to maintain the actual activity people are being referred to”
	Referrals restricted to local funding	“Referrals are only possible within a localized social prescribing remit due to the funding of the referrers”
	Delivery organizations not funded	“We are being socially prescribed by the NHS, but we are not funded by the NHS”

### 3.1 Responses to the survey questions

*Q1:* How does your work involve social prescribing?

Projects worked in some of the UK’s most deprived areas where many had been delivering social prescribing focused on the wider social determinants of health for several years. In addition to delivery, projects mapped community assets and social prescribing pathways particularly for underserved groups. One project collected stories from people of how they had tried to navigate local health systems. A project working with people in recovery and, in some cases experiencing homelessness, used a place-based approach to understand “how community assets, policy makers, researchers, people with lived experience, health and third sectors” could play a role in “the strengthening and integration of community assets, social prescription and civic engagement for people who are often excluded from the system.” A theater-based intervention in Yorkshire working in partnership with a peer support service set up sessions for adults diagnosed with dementia and their carers to engage in creative and cultural activities involving drama, art, poetry, movement and music.

A program for young people accessing child and adolescent mental health services (CAMHS) in the North West provided a social prescribing service where participants were matched with creative practitioners and could take part in ten one-to-one creative sessions of activities such as animation, costume design, photography, print making and song writing. This approach enabled young people to engage in “non-clinical, strengths-based activities that built confidence and supported identity formation beyond diagnosed illness.” A community embedded researcher working with rural communities in the Scottish Highlands described the referral process as “getting their information out to people who they think would really benefit from our work and having them come along to sessions.” Themed sessions, such as a winter theme about presents and warm clothing, took place every 6–8 weeks. People with mild mental health conditions and experiencing social isolation were referred by general and mental health practitioners and drug and alcohol, and community psychiatric nurses and, also, self-referred after finding out about the sessions through word of mouth.

Several projects involved green social prescribing originating from a direct response to community needs; one project noting that “nature should be accessible, bringing people health and social cohesion.” A project prescribing cultural and green inclusion to address inequalities in mental health advised that “ethnic minority and refugee communities in England tend to live in unhealthy urban environments.” The project found that cultural and green assets such as parks, commons, river walkways, places of worship, museums, and community arts groups “have the potential to support mental health and wellbeing,” and that cultural and green engagement can offer “both preventative benefits and management of symptoms of mild to moderate mental illness” yet engagement is “lower among ethnic minority and refugee communities than other communities.” The PI of a green social prescribing project addressing mental health explained that “whilst this region includes some of the least deprived boroughs in London there are pockets of deprivation where minority ethnic, refugee and migrant communities predominantly reside.” Knowledge generated from lived experience workshops with community voice champions and interviews with link workers was used to co-design interventions with community members to “promote engagement with green and cultural assets through improved social prescribing to reduce mental health inequalities.”

*Q2*: Please outline any social prescribing successes you have experienced and how these came about.

Many of the successes described were attributed to link workers. Successes included universities working closely with link workers and community organizations to translate health messages into community accessible languages. Part of working closely with link workers was inviting them to attend hyper-localized team meetings on a regular basis though the ability to attend depended on their case load. Link workers were recognized for “providing a beneficial role as knowledge brokers by collecting information on local organizations and activities and sharing this with clients.” Link workers highlighted the benefits of locating social prescribing in community centers rather than general practices as “many clients, especially young people, struggled to engage with and understand social prescribing based in surgeries, associating the spaces with clinical problems.” Community-based social prescribing was judged as having greater success because “the more informal environment enabled a relaxed atmosphere and the building of understanding and trust.” Another success that projects identified involved link workers providing a helping hand for clients when accessing a community asset through a referral for the first time. This mediation role provided reassurance and support for participants and encouraged attendance. One link worker, for example, accompanied a client normally isolated in their own home, to the local park at the start of a 6-week intervention.

Other successes were attributed to partnerships with primary and secondary care and arts and community organizations, and their roles in co-producing local interventions. A community researcher described their relationship with a general practice as a “massive success” as most of the people who attended the sessions were referred by the general practitioner (GP), adding that “because now there are also drug and alcohol nurses involved, they are prescribing women into our women’s sessions as well.” Another researcher expressed that with social prescribing they were able to reach people that they would not normally have access to and that they could “point to at least four or five people that would never have come to our sessions if they were not ordered to by their GP,” because “when a GP tells you to do something, then people want to do it.” This example highlines the public perception of clinical authority in relation to community-based approaches suggesting a strong trust in individual medical expertise despite dissatisfaction with aspects of the NHS such as waiting times. In a program for young people at a children’s hospital in the North West, co-investigators noted the successes as “significant improvements in confidence, wellbeing, and re-engagement with everyday life” and that young people reported feeling “more positive, more socially connected, and more able to pursue personal interests.” Feedback also highlighted that participants returned to mainstream education, reconnected with family, joined community groups, and built new friendships after completing the program.

In the Scottish Lowlands, bringing practitioners from the health and care sector together with people in recovery for three workshops was successful in co-designing a six-week intervention in a community garden and developing trusted relationships. In an area with one of the highest male suicide rates, over an 18-month period 23 men stated that green social prescribing offered alongside clinical treatments prevented them from taking their own lives. For a project in Yorkshire, success included forming “cultural partnerships” with organizations to create more accessible routes for people with dementia to engage with cultural offers. A researcher from the project explained that “people who have never been to the theater or museums have had a new world opened up to them with careful, dedicated support.” Partnership working was used to engage people with dementia in co-creating a theater festival of dementia and hope where “individuals with dementia took creative leadership roles in planning the festival.” Working closely with delivery partners and embedding creative health offers within place-based organizations while mapping the different routes and referral pathways was deemed successful for working with young people. Another success was forming a new choir for people living with dementia; through social care referral, “people who felt singing was not for them have been supported to try it out and have loved it.”

Further successes included creative methodologies and approaches to evaluation that involved ethnographic and video observation with people in recovery or experiencing homelessness, photovoice and other participatory methods with people with severe mental illness, and creative workshops and questionnaires with health and social care practitioners. In addition to ethnographic and art-based methods, a minority of projects successfully used the Warwick-Edinburgh Mental Wellbeing Scale (22) to report outcomes. Another success was collecting the stories of people navigating their local health systems to identify and address gaps needed for effective social prescribing providing a “better understanding of a new system to connect individuals from healthcare programs to community-based opportunities.”

*Q3*: Please outline any social prescribing challenges you have experienced and how these were addressed.

In addition to successes many of the challenges concerned link workers, for example, their line management and how their case load and flexibility to engage in creative approaches varied depending on whether they were managed through primary care or another organization. Projects reported a lack of knowledge among link workers about “what is available in terms of support from community assets” with “unknown pathways” that might “restrict access to services for people experiencing diverse vulnerabilities.” One project identified that a key challenge was the lack of integration of the “local asset ecosystem into the ways of working of the social prescribing workforce.” Although link workers were aware of the benefits of integrating cultural and green community assets into the referral process, particularly to address social isolation and mild to moderate mental health issues, low awareness of existing community assets reduced the referral options. Furthermore, link workers found that integrating assets sustainably and in an appropriate way to meet the mental health needs of marginalized and underserved communities was challenging. There was instead a tendency of link workers to focus on referrals to deal with “more pressing needs” such as housing or debt and although this seemed a reasonable stance given that these needs form part of the wider determinants of health, the demand for such services often precluded them from incorporating local assets into their role. To address these challenges, projects formed partnerships between the universities, NHS, policy makers, and non-governmental organizations. One partnership participated in a reflexive mapping exercise to develop new social prescribing pathways.

A major challenge for green social prescribing was accessing suitable venues, as allotments and other local green spaces did not feel accessible for non-English speakers or newcomers to the area. Furthermore, “local authority rules discouraged communities to use and take ownership of green spaces for health.” Preliminary research indicated though that both issues might be addressed through closer partnership working. For example, a project in the West Midlands reported working with closely with stakeholders to map community assets “as communities see them” and described the move away from traditional sports grounds to other less utilized spaces. A representative from a third sector organization involved with a different project explained that space was a challenge because as they were “getting so many referrals and people now,” they needed to “borrow someone’s land” to hold the sessions and “if they are doing something on the day, we have to move out of the way.” They continued to elucidate that “because there is loads of us now, and we are expecting more if other doctors in the surgery start prescribing, I’m thinking, what are we going to do with all the people?”.

One project focused on the challenges of scaling up and delivering social prescribing in remote and rural areas, outlined many challenging factors, also typical of other projects, including geographical and logistical issues; accessibility and lack of public transport; low numbers of referees; variable referral pathways; negative perception of the heritage sector; sector sustainability; volunteer workforce recruitment and retention; and training and support requirements. Additional challenges experienced by another project included “stigma associated with mental health among some ethnic minority and refugee communities” and a “poor understanding of the benefits of engaging with community assets.” To counter this issue, this project became involved in the co-design of interventions to promote the inclusion of community assets in social prescribing. A PI from a different project described another challenge: community members came to them in the hope that they could solve everything “because they are being bounced from pillar to post and coming up against broken systems and closed doors” but much of what people were hoping for was “outside of our power.” They continued to express that “people just want so much because they need so much, and we quite often have to say actually that is not our remit; you have to know where those boundaries are.”

Two survey respondents expressed issues with attendance; a council member recalled a message from their partners saying that they had “so many community members in mind who would really benefit.” They continued to reflect, however, that “they just did not turn up.” When they spoke to the group, the reason for not attending was “because there was research attached to it.” Additionally, people were unsure of the council involvement, asking “what does the council research, and what do they want to know about me?” This hesitancy was especially true of “people who are already unsure of systems and new places and people” such as migrants to the area. The other respondent articulated the challenge that “many young people face significant personal, emotional, and structural barriers that limit participation” and that non-attendance was “often linked to anxiety, depression, or low motivation which can make leaving the house difficult.” The project used strategies such as introductory videos from practitioners to help reduce the fear of meeting someone for the first time.

A further challenge was funding; although projects completing the survey had received Mobilizing Community Assets program awards so were not responding with a view to gaining further funding for themselves, several researchers commented on the availability of funding for research but not for service delivery. A researcher from one project, for example, outlined the work of an arts organization being “socially prescribed by the NHS but not funded by the NHS,” writing further “it was wonderful that social prescribing was going so well” but it was not “backed up with any money.” The researcher described how the NHS referred patients to third sector organizations that were “scrambling to keep their heads above water” and that money “was always the challenge.” Funding was also described as “always an issue for arts organizations… to maintain the actual activity people are being referred to.” Another researcher referred to an arts organization they were working with which had expressed that their offer attracted interest from people in a broader geographical area, but referrals were only possible within a localized social prescribing remit due to the funding restrictions of the referrers. Restricted funding also led to programs of sessions being relatively short. A community researcher in the Scottish Highlands found that referral to six-to-eight-week programs or drop-in sessions were unsatisfactory for most participants, writing “what we are learning is that that does not really work for people, they want to be prescribed into something and then go on a really long journey.” Despite the cost of provision, projects expressed the importance of prescribed interventions being free of charge maintaining that “the referral service and creative activity have been free to the participant” and it “is crucial for people from marginalized or lower socio-economic backgrounds, as financial barriers do not arise at the point when people are feeling most vulnerable or ill at ease.” One project explained that as financial barriers were significant for some families, a charity associated with a children’s hospital in the North West covered transport costs for parents to reduce the inequity of access.

*Q4*: To what extent do you think that your social prescribing work is tackling health inequalities?

Many projects expressed the belief that their organizational commitment and leadership was “centered around tackling health inequalities at the root,” that convening communities produced “support from within” and referred to working “in a hyper-localized way.” Projects stated that their work was inspired by Sir Michael Marmot’s wider social determinants of health and “underpinned by the Marmot principles” (7, 9, 23). Using place-based approaches, projects developed an understanding of the role that community assets play to improve general health through social prescribing and civic engagement by focusing on preventative health, such as cancer screening and dementia. Other projects reported generating knowledge through interviews, workshops, focus groups, and community walks used to co-design interventions with community volunteers and link workers to promote cultural and green community assets. In one case, interventions were used to address ethnic inequalities in mental health. Co-investigators from a different project tackled health inequalities by examining arts-based therapies to support the mental health and wellbeing of young people underserved by formal mental health pathways; they described their participants as facing “overlapping disadvantages, including financial hardship, disrupted education, social isolation, and long waits for formal services.” They explained how frontline workers reduced inequality by offering “early relational support to young people who may not meet specialist thresholds but are still in need of support.” At a wider level they felt that they were “helping to address systemic inequalities by improving consistency, coordination, and visibility of creative health provision across the North West.” Rather than being limited to one locality, the role supported “cross-area learning, resource sharing, and development of a shared language, supporting organizations that might otherwise operate in isolation.”

A project working with migrants and refugees addressed health inequalities by holding welcome sessions co-designed with their partners and other agencies. In response, one migrant looking back on their entry to the UK observed that “if we had been able to access welcome sessions that were creative and could build community, then we could have made links into lots of other services and that would made a difference to our lives.” Aware of the idea of research being off-putting to target audiences, a PI explained that they “made it very clear to people from the get-go, that we research health inequalities through on-the-ground, co-participatory work. I think because it is so obvious and there is nothing hidden about it at all, that makes it easier.” A community researcher from another project expressed uncertainty as to whether they were tackling health inequalities, writing “We’re having people share that they are coming off antidepressants, and people just feeling better in themselves, it is massively tackling health issues,” continuing with “whether it is tackling health inequalities is another thing entirely… it is certainly highlighting them” and carrying on to express “I think we are giving people the strength and confidence to be able to deal with their own lives and then they can tackle their own inequalities.”

## 4 Discussion

Projects from across the three phases of the Mobilizing Community Assets program aimed to promote health and wellbeing and tackle health inequalities by addressing the social and behavioral determinants of mental and physical health and using social prescribing to achieve these aims. In accordance with current findings, the Commission on the Social Determinants of Health advised that health inequalities were associated with an “unequal distribution of power, income, goods, and services, globally and nationally” leading to “unfairness in the immediate, visible circumstances of people’s lives” [(6), p. 1]. In keeping with the program aims, a Delphi study to determine an accepted definition of social prescribing (15) recognized the ways in which “social prescribing relates to the social determinants of health, health equity and non-medical, health-related social needs” [(15). p. 8]. Projects used hyper-local place-based, co-design and creative approaches to illustrate how partnerships between arts, community, and health and social care organizations, link workers, local authorities, peer support groups, people with lived experience, policy makers, volunteers, and universities could play a greater role in strengthening and integrating community assets within social prescribing (24). Survey responses showed that projects worked in the UK’s most deprived areas delivering social prescribing focusing on the social determinants of health, and mapping community assets and pathways for underserved groups. Furthermore, projects found that people experiencing the worst health outcomes were often excluded from health and care systems. Although the research implication that better community mapping helps to improve services is broadly true, there is an issue here in that mapping assets too rigorously could counteract their otherwise organic ethos, especially for tackling sensitive issues, consequently, trust becomes a prominent factor in community-based initiatives.

The 10 year health plan for England made proposals that align with the aims and objectives of social prescribing and community-based initiatives in the Mobilizing Community Assets program. These proposals concern tackling health inequalities, care in the community, and partnership and collaboration. The plan unfortunately failed to mention the role of creative health or the wider cultural sector despite evidence and lobbying (25). Nevertheless, projects surveyed agreed with the necessity of health prevention seen by the plan as key to tackling health inequalities, whilst also boosting the labor market and reducing NHS costs (14). Several of the projects took an active role in primary prevention, health prevention and healthcare promotion though none involved secondary prevention comprising genomics, predictive analytics and artificial intelligence to pre-empt need and risk. The plan’s emphasis on health prevention is consistent with a conceptual definition of social prescribing which referred to the “central tenets of health promotion and disease prevention” and which offered “a way to mitigate the impacts of adverse social determinants of health and health inequities by addressing non-medical, health-related social needs” [(15), p. 8].

Factors understood to be responsible for influencing inequalities embrace early childhood, education, occupation, income, gender, and ethnicity, underpinned by social and economic elements including “material circumstances, the social environment, psychosocial factors, behaviors, and biological factors” [(9), p. 16]. Social and economic elements include issues such as housing, food, employment, income, and social support (15). Respondents in the current study, however, regarded link workers dealing with what they saw as more pressing social needs, such as housing or debt, as a challenge, as these issues distracted from referral to cultural and green interventions. As it seems logical that link workers would want to resolve the immediate requirements of their clients, this type of comment by respondents may reflect a bias toward their own offer or field of research and represent a tension between the role that link workers might instinctively fulfil, and the role that others might assume they should be addressing. It seems likely, however, that this tension was not seen as intractable because respondents wrote about addressing challenges of this sort through closer partnership working and, over time, developing a greater degree of trust in relationships.

The purpose of the 10 year health plan’s proposal to move care into the community was to deliver value-based services that are continuous, accessible, and integrated with proportionately greater investment in out-of-hospital treatments (14). To achieve this objective, the plan recommended establishing Neighborhood Health Centers, initially in areas of lowest life expectancy, to house multi-disciplinary teams and offer a single entry point for patients. This collaboration of multiple disciplines is one which Mobilizing Community Assets has consistently promoted, particularly with respect to co-location of NHS, local authority and voluntary sector services, in addition to partnerships with commercial partners, local councils, and universities. Co-location can be difficult to achieve as trusting relationships between partners need to be developed over time although developing a typology of community-based activities and assets, under an overarching social determination framework can contribute greatly toward understanding how to organize services, to make social prescribing more efficient and effective. Given that the National Director of Patient Experience within the Department of Health and Social Care advised disbandment of Healthwatch England and local Healthwatch groups, this action raised questions about the presence of an independent patient and community voice to hold the NHS to account (26). Another issue is that the plan appears to be the shift away from the voluntary, community, faith, and social enterprise (VCFSE) sector (26) shown to be instrumental in at least two thirds of social prescribing projects surveyed. The local VCFSE sector will be limited to individual systems and places, whilst the proposed reduction of integrated care systems will add further disruption (26). The plan instead involves bringing more integrated services into local communities to give patients greater autonomy in tailoring care to their individual needs, obtaining care in their own neighborhoods and having access to their medical notes (14).

Offering patients greater autonomy fulfils an innate psychological need expounded by self-determination theory along with the needs of competence and relatedness that when achieved, maximize wellbeing and motivation (18). The theory emphasizes the importance of both motivational quality and quantity, critical in initiatives that require patients to take responsibility for their own health and wellbeing, and essential for both mental and physical health improvement (19). A meta-analysis of self-determination theory in healthcare contexts found that that behavior change was more effective and long lasting when participants were autonomously motivated (19). There was no way to determine whether behavior change was longer lasting in the current study because although most projects surveyed had been running for several years, none appeared to have conducted robust longitudinal evaluation. The results of the meta-analysis indicated that autonomy and autonomy support, such as by a GP or link workers, are predictors of healthy behavior and psychological wellbeing (19). Furthermore, the concept of autonomy is important in medical ethics which concern respecting the wishes of individuals (19), such as the right to refuse treatment. The authors suggested that public health policy makers might include the principles of self-determination theory in delivering their messages to promote health living (19). Findings from the current study imply that creative interventions particularly in cultural, natural or heritage environments, encouraged autonomy where participants were able to take control over their own actions and make independent decisions especially important for recovery, especially for mental health and wellbeing.

In keeping with models of recovery (16), four projects employed peer support groups for people with dementia; people in recovery and/or experiencing homelessness, mental health referrals; and preventative health care screening. In these projects, peer support workers used their lived experience of a particular issue to offer support to people in a similar position. In three other projects, people with lived experience were instrumental in supporting and advising on projects for mental health including green community solutions, creative activities for children and young people, and enabling access to green spaces for underserved groups. Evidence has shown that green social prescribing in natural environments can be effective for mental and physical health and supporting children and young people (27–29). As part of peer support relationships, the community reinforcement approach demonstrated the importance of connection with others in maintaining recovery (16) and a degree of competence over coping with adverse experiences, consistent with self-determination theory (18). Although traditional models of recovery use a 12-step intervention (30), there has been a rise in the adoption of alternative forms of peer support within treatment and community settings (16), as in the projects reported here, because of the potential benefits offered to patients (31). One of the issues with peer support, however, is that it has not been rigorously tested consequently it is difficult to determine its effects (32). A literature review of studies of peer support demonstrated associated benefits such as higher rates of abstinence and more satisfaction with the treatment but also showed a lack of rigorous empirical studies (16). In the current study projects assumed that tried and tested methods such as support from peers and people with lived experience would be beneficial, however no evaluation was carried out as to its effectiveness.

### 4.1 Limitations

Surveys were conducted with research leads working on a range of social prescribing projects including cultural, heritage and nature-based activities in different UK locations. Although respondents appeared reasonably representative of the research program, the selection of responses they provided may not necessarily have been typical of UK social prescribing practices as a whole; this sampling bias implies that it may be difficult to generalize the findings beyond that of the research program. Furthermore, survey design inherently possesses limitations arising from self-reported information, such as social desirability and recall biases, that can compromise the quality, accuracy, and validity of the data. Survey responses varied with some respondents making either general comments or those pertinent to specific projects, such as access in rural areas. Furthermore, the phase under which a project was funded may have acted to steer responses in a specific direction leading to more minority views. For example, phase one respondents referred to community partnerships and asset mapping whereas those in phase two emphasized cross-sector and collaborative approaches and in phase three, concentrated in scaling up place-based community projects. The fact that four projects took part in two phases (either one and three, or two and three) made it difficult, however, to tease out any effects of patterns or trends related to phase. Future research might consider using follow-up interviews to ask further questions addressing differences in project approaches across the three phases. Conversely, responses for which there was consensus, evidenced in the themes, increased generalizability of the findings, and impact and reach of the research. A further limitation was that although “successes” and “challenges” are widely used terms, they could not be clearly delineated in that once a challenge is overcome, it might become a success. For the current research, identification of whether outcomes were successes or challenges was based upon respondents’ interpretations. To minimize bias, rigorous analytical techniques were used for the thematic analysis to produce trustworthy data (21) however reflexivity in the researcher’s role and its potential influence on the research process should be acknowledged. In employing an interpretivist epistemology, the researchers recognize they were never removed from the research process and acknowledge that their understanding may have predisposed them to certain assumptions. Due to the limitations, broad consensus about conclusions regarding social prescribing should be treated with caution.

## 5 Conclusion

In addition to the self-management of care and clinical treatments, social prescribing has been used to engage communities and to build trust, helping to increase the number of years lived in good health and redress the balance of health across the UK. With policy focused on prevention and re-location of care into communities to reduce the burden on acute services (14), the need for robust evidence from social prescribing schemes that integrate community assets into health and care systems is becoming increasingly important. The research indicates that evidence-based models of social prescribing which reliably integrate community assets into health and care systems are essential for future care provision. A consortium-based approach to provision is needed, working closely with local populations and public health to target under-served communities. Key success factors comprise co-production, trust and flexibility particularly within partnership working. Implementing social prescribing involves creating a bridge between healthcare providers and community assets to address the biopsychosocial and practical needs of clients. The implementation process should be structured into steps comprising identification of the target community and needs assessment; mapping community assets; identifying stakeholders such as GPs, community leaders and the VCFSE sector; assembling a core team; and developing a structured implementation plan that defines the role of link workers, the referral pathway and what the service will and will not offer. Workforce competencies for link workers should focus on individualized care, active listening, empathy, and building trust to connect individuals with community assets, following the workforce development framework (33).

It is important to stress that social prescribing models will need to be carefully and collaboratively set up with sufficient long-term funding, particularly to sustain link worker roles after project completion as posts are frequently funded through time-limited grants rather than core statutory budgets. Models also need to be developed with the potential to be scaled across a broader geography and to other regions with robust means of evaluation, particularly longitudinal assessment, and governance structures in place from the onset. Effective governance structures for social prescribing require a collaborative, whole system approach that combines clinical safety with community-led, VCFSE sector expertise. The framework needs to move away from traditionally rigid hierarchies toward shared leadership, enabling partners to co-design and manage services, and ensuring that decision-making is rooted in community needs. Other recommendations include the need for stronger collaboration across the VCFSE providers as they can play a key role in gathering and disseminating community needs and voice especially given the disbandment of Healthwatch. The nature of the provision should be determined by consortia in conjunction with relevant data concerning health conditions and the social determinants of health including socio-demographic and socio-economic drivers such as employability and housing conditions. All types of provision, whether creative, nature-based, sports, social, or legal, should be co-located wherever possible and co-delivered to address the root causes of social inequity, health exclusion, and wider social determinants. Furthermore, and in keeping with the 10 year health plan, treatment and prevention need to be person-centered and across co-morbidities and multiple determinants of health to fully tackle health inequalities.

## Data Availability

The raw data supporting the conclusions of this article will be made available by the authors, without undue reservation.
